# Supplementary material for: Prevalence, Risk Factors of Preserved Ratio Impaired Spirometry in adult in plateau: A Cross-Sectional Study
Source: PLoS One. 2025 Apr 10;20(4):e0318546. doi: 10.1371/journal.pone.0318546 (PMC12026938; doi:10.1371/journal.pone.0318546)
Supplement: Attachment — Submitted filename: Response to Reviewers.doc [file pone.0318546.s002.doc]

**Answers to reviewers**

Changes in the main text indicated in red

Reviewer 1:

1. *In introduction: The introduction should clearly state the need for the study.*

Thank you very much for recognition of our work. We really appreciated this advice.

COPD is a major public health concern, and PRISm is currently considered to be pre-COPD, which can progress to COPD in some people over time. The incidence of COPD is higher in plateau areas, especially in Asia. Therefore, understanding the prevalence and risk factors of PRISm in highland areas is helpful to prevent COPD.

2. *In Page No-2, line-50: “Studies1 found that the prevalence of COPD was higher in high-altitude areas.” At least 2 or more than 2 citations should be quoted for'studies’.*

We really appreciated this advice; We added two more citations for a higher incidence of COPD in high areas.

3. *In Page No-3, line-57: “Previous studies have shown that individuals with 58 PRISm…….4,5,6” –It would be good if authors included* *the place of study for these references.*

We really appreciated this advice; We added the place of study.

4. *In Page No-4, line-85: “According the guideline, we used the survey used uniform methods, procedures….”. – It would be good if the authors state the name of the guideline in the manuscript.*

We really appreciated this advice; We added the name of the guideline.

*5.In Page No-6, line-157: “This study was consistent with most previous studies.” –* *The references should be added for most previous studies..*

We really appreciated this advice; The references included references 9 and 16

Reviewer 2:

*1.The study relies on self-reported data from participants for key variables like smoking history, history of tuberculosis, and medical conditions (diabetes, hypertension). This introduces potential recall bias, where participants may inaccurately report their histories. This could especially affect data related to smoking habits and tuberculosis history, as both factors have strong social and stigma-related influences.*

We really appreciated this advice*.*Our questionnaire was conducted in a quiet room by a trained respiratory physician. All patient information is kept confidential and is asked based on a standardized questionnaire. We also reviewed the participants' medical records. This reduces recall bias to some extent. However, due to the backward medical conditions in Tibetan areas on the plateau, many participants' medical records are incomplete, and some patients' medical records in other places cannot be provided, which will indeed lead to recall bias. Thank you very much for the comments. In the future work, we will pay more attention to this problem and make the obtained data more authentic and credible.

2. *Although the study briefly mentions different prevalence rates among ethnic groups (Han and Tibetan), it doesn’t explore whether these differences are statistically significant or delve into the possible cultural or genetic factors influencing these rates.*

We really appreciated this advice*.*In the single factor analysis, we found that the prevalence of different ethnic groups was inconsistent, but after the multi-factor analysis, it was found that ethnicity was not an independent risk factor. That's why we didn't talk about nationalities.

Reviewer 3:

1. *Sample size calculation and power analysis were not done.*

We really appreciated this advice. Since there is no available research data for reference in China, we use a single proportional formula to calculate the sample size. N=Z2*P (1-P) /d2. Then the sample size is 544 based on the total population. Considering that the loss of follow-up rate was 10%, the final sample size was 560.

2. *I can not find the tables (table 1 and table 2) to evaluate the results.*

We really appreciated this advice.We have put Table 1 and Table 2 in the manuscript.
