# Supplementary material for: Prevalence, Risk Factors of Preserved Ratio Impaired Spirometry in adult in plateau: A Cross-Sectional Study
Source: PLoS One. 2025 Apr 10;20(4):e0318546. doi: 10.1371/journal.pone.0318546 (PMC12026938; doi:10.1371/journal.pone.0318546)
Supplement: Attachment — Submitted filename: Response_to_Reviewers_auresp_2.doc [file pone.0318546.s003.doc]

**Answers to reviewers**

Changes in the main text indicated in red

Reviewer 2:

1. *-Conduct Power Analysis:Include a detailed power analysis to support the adequacy of the sample size.*

Thank you very much for recognition of our work. We really appreciated this advice.

This is a cross-sectional study,since there was no available research data for reference in China, we used a single proportional formula to calculate the sample size. N=Z^2^*P (1-P) /d^2^. Then the sample size was 544 based on the total population. Considering that the loss of follow-up rate was 10%, the final sample size was 560(Z=1.96,p=0.15,d=0.2p).

2. *Address Recall Bias:Highlight steps taken to minimize recall bias more explicitly and suggest future improvements.*

We really appreciated this advice.Our questionnaire was conducted in a quiet room by a trained respiratory physician. The uniformly trained respiratory physicians asked the respondents face to face and asked questions in the independent and quiet room according to the questionnaire items.At the same time, we reviewed the respondents' medical records. If there was any discrepancy between the medical records and the responses, the medical records were taken as the reference.

3. *Explore Ethnic Variations:Delve deeper into the differences observed between ethnic groups, providing statistical significance and possible explanations.*

We really appreciated this advice*.*Our previous research has found that the prevalence rate of COPD in Han population was higher than that in Tibetan population, suggesting that the prevalence of COPD may be related to ethnic and racial differences. Havryk et al studied Sherpas living at an average altitude of 4000 meters, showing that their lung capacity increased by about 12% compared to Caucasians. Wood conducted lung function studies on Ladakh and Tibetans living between 3300 and 4500 meters above sea level. Spirometry results in both groups showed high values of maximal mid-expiratory ﬂow are between 130% and 150% of predicted, and a FEV1/FVC ratio of 115%. These studies suggest that, over time and over multiple generations, highaltitude populations have undergone physiological and genetic adaptations to extreme altitudes in response to chronic hypoxia and generally high-intensity exercise. Living at high altitudes is associated with accelerated lung function decline in populations with low daily smoking rates. However, the lung function of the Han population is lower than that of the Tibetan population, and is more susceptible to decline due to environmental factors.However, In this study, We found no statistically significant difference in prevalence among different ethnic groups. To find out why, prospective long-term follow-up studies may be needed.

*4.Enhance Discussion:Expand the discussion to include comparisons with global studies and implications for public health policy.*

We really appreciated this advice. At present, there is a lack of global data on PRISm studies in plateau areas, and the results of this study are similar to those of other studies, which found that smoking, age, BMI≥30, diabetes, history of tuberculosis, hypertension, white blood cell count, and red blood cell volume distribution width are correlated with PRISm. The purpose of this study is to supplement some of the plateau data for the global PRISm research.In highland areas, we can prevent PRISm development by controlling weight, smoking, diabetes, and high blood pressure. A history of tuberculosis may be a specific risk factor for PRISm in the plateau region of China. We can also detect PRISm early by focusing on the WBC and RDW of the population.

*5.Improve Data Accessibility:Consider anonymizing the dataset to allow partial access for peer verification while adhering to ethical guidelines.*

We really appreciated this advice.Ethics was approved by The Third Hospital of Mianyang's Ethics Committee. As part of the ethics review process, participant confidentiality restrictions prohibit the authors from making the data set publicly available. During the consent process,participants were explicitly guaranteed that the data would only be seen my members of the study team. For any discussions about the data set please contact The Third Hospital of Mianyang's Research Governance:578012475@qq.com.
